# Safeness and efficacy of 2-µm handheld thulium laser during microsurgical resection of supratentorial and infratentorial meningiomas: Experience of a single center

**DOI:** 10.3389/fsurg.2022.1021019

**Published:** 2022-12-16

**Authors:** Julio Alberto Andrés Sanz, Salvatore Marrone, Guglielmo Cacciotti, Ettore Carpineta, Carlo Giacobbo Scavo, Raffaele Roperto, Domenico G. Iacopino, Rinat Sufianov, Aidar Safarov, Albert Sufianov, Luciano Mastronardi

**Affiliations:** ^1^ Department of Neurosurgery, San Filippo Neri Hospital/ASLRoma1, Rome, Italy; ^2^ Department of Neurosurgery, Lozano Blesa Clinical Hospital, Zaragoza, Spain; ^3^ Neurosurgical Clinic, AOUP Paolo Giaccone, Department of Biomedicine Neurosciences and Advanced Diagnostics, University of Palermo, Palermo, Italy; ^4^ Department of Neurosurgery, The State Education Institution of Higher Professional Training, The First Sechenov Moscow State Medical University Under Ministry of Health, Moscow, Russia; ^5^ Federal Centre of Neurosurgery, Ministry of Health, Tyunmen, Russia

**Keywords:** 2-µm thulium laser, intracranial meningioma, microsurgery, resection, tool

## Abstract

**Aims:**

We performed a retrospective nonrandomized study to analyze the results of a microsurgery of intracranial meningiomas using 2-μm thulium flexible handheld laser fiber (Revolix jr).

**Methods:**

From February 2014 to December 2021, 75 nonconsecutive patients suffering from intracranial meningiomas, admitted in our department, have been operated on with microsurgical technique assisted by 2-μm thulium flexible handheld laser. We have reviewed demographic and clinical data to evaluate safety and efficacy of the technique.

**Results:**

There were no complications related to the use of the 2-μm thulium laser. We operated on a high percentage of cranial base and tentorial and posterior fossa meningioma in our series. The neurological outcome and degree of resection did not differ from previous series. The neurosurgical team found the laser easy to use and practical for avoiding bleeding and traction.

**Conclusion:**

The use of 2-μm thulium fiber handheld flexible laser in microsurgery of intracranial meningiomas seems to be safe and to facilitate tumor resection, especially in “difficult” conditions (e.g., deep seated, highly vascularized, and hard tumors). Even if in this limited retrospective trial the good functional outcome following conventional microsurgery had not further improved, nor the surgical time was reduced by laser, focusing its use on “difficult” (large and vascularized) cases may lead to different results in the future.

## Introduction

Intracranial meningiomas can be grossly distinguished into supratentorial and infratentorial locations. Regarding its treatment, the approach depends on the case: wait and watch is an option for small cases or nongrowing tumors; stereotactic radiosurgery (SRS) has been advocated as a growth-control therapy, with relatively good results. Also, endovascular embolization plays its role, mainly as a surgical adjuvant. However, surgery remains the only curative treatment (1).

For those meningioma emerging from the cranial base, depending on the tentorium or occupying the cerebellopontine angle (CPA), it is mandatory to avoid injury to the capital surrounding structures (main intracranial vessels, cranial nerves) and limit traction from brainstem structures, which could cause microvascular disruption and major morbidity. Thus, a necessity for innovative tools emerges, which could improve outcomes and reduce risks in complex meningioma surgery.

Laser surgery has the advantage of reducing mechanical trauma and intraoperative bleeding, valuable characteristics for meningioma resection. The first use of lasers in neurosurgery dates back to 1966 (2, 3). Interest in them has been fluctuating throughout history because of the lack of refinement of the initial laser technologies, which implied large equipment and some troublesome characteristics. However, this technology has been improved, and several models were developed. Initial pulsed-wave lasers were largely replaced by continuous-wave lasers (4), capable of delivering focused and nondispersive energy to dissect and vaporize histological targets. Another significant landmark was its presentation as a handheld fiber since the development of the flexible CO_2_ laser in 2005 (5).

**Figure 1:**
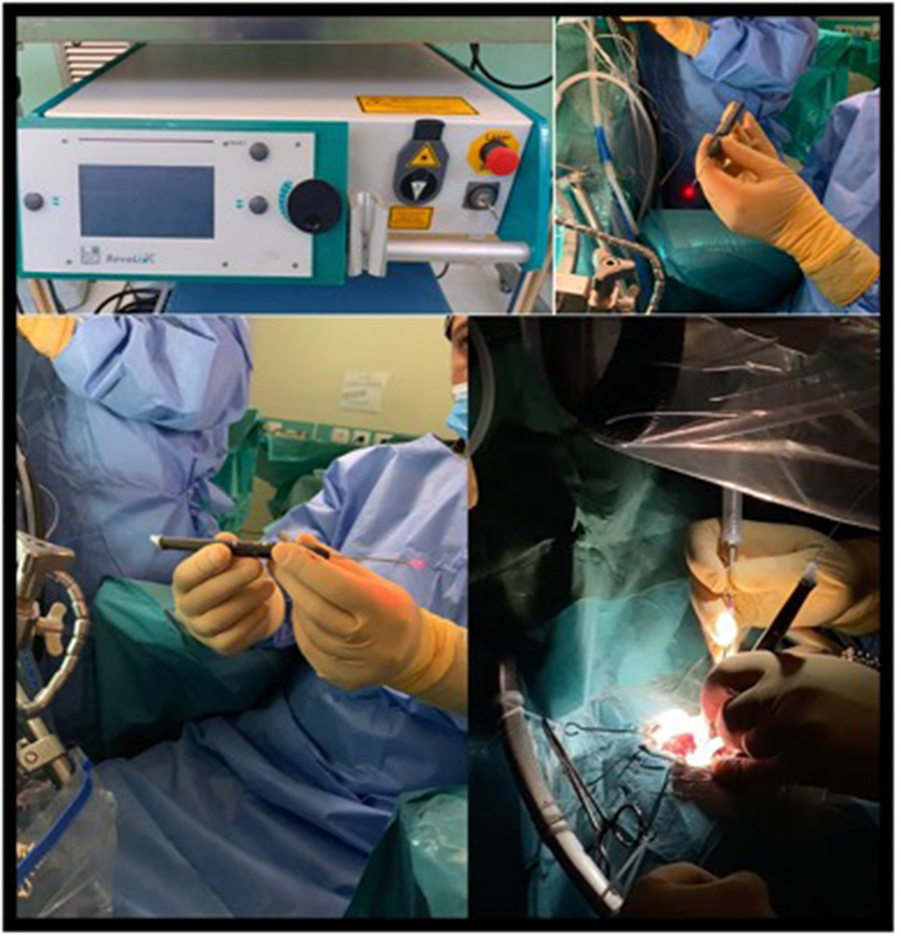
The 2-µm thulium laser device. Top left: console. Top right and bottom left: handheld device showed by GC. Bottom right, pic during active using on the surgical field. Among its features, we underline the compact presentation, which requires little space in the OR, and the transmission through optic fiber, which can fit in handheld and endoscopic terminals.

Since 2015, the 2-μm thulium laser has been used in medicine, thanks to its promising characteristics (6). Although initially developed for urological surgery, recently it has been adopted for neurosurgical procedures. It is capable of delivering focused and nondispersive energy to dissect and vaporize histological targets, proving coagulation and cutting capacities. The equipment consists of a standard size console connected to an optical fiber cable that transmits the wave. This fiber can be then inserted in different terminals: a handheld piece or an endoscope (Figure 1). When required, the laser is activated by a pedal switch, by the operator, who handles the terminal and directs it toward its objective. Distance of application will depend on the desired effect: closer for cutting, slightly further for superficial coagulation, but will range approximately between 2 and 10 mm. The device does not pose space problems in the operating room, has no particular maintenance or refrigeration needs, and offers easy and intuitive operation.

Its appliance has already been described for endoscopic third ventriculostomy as well as for schwannoma microsurgery (7, 8). Some initial reports showed its feasibility for meningioma surgery (9, 10). Our group began using it in 2014, particularly for resection of cranial base and posterior fossa meningiomas.

This paper intends to depict our experience with the first 75 cases operated on with this tool, which constitutes the largest series up to the date in the literature.

## Materials and methods

### Patient data

Seventy-five nonconsecutive patients with intracranial meningioma were selected for this retrospective review. Inclusion criteria were as follows: anatomopathological diagnosis of meningioma, cranial or craniospinal location, age 18 or older, and undergone surgical treatment. In our series, all the patients were operated on by microsurgery techniques combined with the assistance of a thulium-based flexible handheld laser fiber. Demographic and outcome data were collected and stored in a database using Excel, and analyzed retrospectively.

### Diagnosis and determination of tumor locations

Each patient received an MRI scan not exceeding 1 month preoperatively. The tumors were measured in three spatial dimensions (on axial and coronal MRI section planes), and their sizes were estimated considering their major diameter, excluding the dural tail.

Because different locations of meningioma have considerable neurosurgical implications, we have distinguished between supratentorial (convexity, falx, medium cranial fossa/sphenoidal, or others), infratentorial (posterior cranial fossa: cerebellopontine angle, petroclival, tentorial, or others), and craniocervical junction meningiomas.

### Flexible 2-μm thulium laser system

In these 75 cases, we utilized handheld 2-μm thulium flexible laser fiber (Revolix jr®, Lisa laser USA, Pleasanton, CA, Unites States) (Figure 1). The range of power settings was 1–14 W. The laser is not hindered in its function by the presence of water, so the standard 0.9% saline solution irrigation is used for cooling the laser fiber. The fiber was used for cutting, vaporizing, and coagulating the capsule and the intracapsular mass, in combination with bipolar forceps, micro scissors, and ultrasound aspirator device. Following debulking, microdissection was performed as usual for tumor capsule detaching from the surrounding tissue. In cases with some dural remnant that could not be removed, thulium laser was used for dural implant coagulation.

### Determination of tumor grade removal, procedure time, and neurological outcome

The amount of tumor removal was determined by the surgeon's impression as well as by postoperative contrast-enhanced MRI performed within 48 h after surgery. We classified the extent of resection using the Simpson scale.

Neurological outcome was classified into two main categories: (a) without new neurological deficit or improvement of the previous one after surgery and (b) with new neurological deficit after surgery. Among these, we distinguished (a) major neurological deficit, (b) cranial nerve deficit, (c) motor deficit, and (d) speech impairment.

## Results

### Demographic and clinical data

This series included 53 females and 22 males (ratio F/M 2.4:1). Ages ranged from 28 to 83 years, with a mean age of 57.8 years and a median of 57 years. We recorded 32 supratentorial lesions, 40 infratentorial tumors, and 3 craniospinal meningiomas.

Among the supratentorial group, F/M ratio was 1.91, mean 62.3 years, and median 63 years. The infratentorial groups were younger, with a mean age of 54.5 years and a median age of 55 years, having the F/M ratio of 2.91.

### Approach and degree of resection

We show our results in Table 1, concerning location, approach, and degree of resection. The individual patient data are shown in Supplementary material. We would underline that it is a series with a high percentage of complex meningioma, settled in cranial base location most of them. All except one (combined approach) was operated by an open approach. Tailored craniotomy or pterional were the preferred option for supratentorial location, whereas retrosigmoid approach was the most frequently used for p-fossa location. In our craniospinal cases, we used the extreme lateral transjugular infracondylar (ELITE) craniotomy (11).

**Table 1:** Results summarized. Aggregated data according to supratentorial or infratentorial location

Supratentorial		Infratentorial	
*N*	32	*N*	43
**Location**			
Middle fossa/sphenoidal	17	CPA	14
Convexity	1	Petroclival/posterior petrous bone	18
Parafalcine	10	Tentorial	5
Others (intraventricular, orbital, tuberculum sellae)	4	Posterior foramen lacerum	
		Foramen magnum	4
**Approach**			
Pterional	12	Retrosigmoid	34
Ad-hoc	13	ELITE	5
Kawase	2	Kawase	2
Orbitopterional	2	Suboccipital	1
Frontobasal	2	Supracerebellar infratentorial	1
Endoscopic	1		
**Degree of resection**			
Simpson I	13	Simpson I	12
Simpson II	9	Simpson II	16
Simpson III	3	Simpson III	6
Simpson IV	7	Simpson IV	9
**Was a reintervention**			
Yes	8	Yes	2
No	24	No	41
**Postoperative deficit**			
No neurological deficit	23	No neurological deficit	28
Motor deficit	3	Cranial nerve deficit	10
Motor + speech deficit	2	Motor + CN deficit	2
Cranial nerve deficit	2	Speech impairment	1
Speech deficit	2	Level of consciousness impairment	2
**Documented to have received**			
Yes	2	Yes	2
No	30	No	41

### Neurological outcome

Aggregate data are also shown in Table 1. It should be noted that neurological status of this series patients was same or better than preintervention in 47 cases (62.7%).

In two cases (2.7%), there was evidence of postop level of consciousness impairment (in those cases, the patients already manifested neurological deterioration at the time of admission). Most of focal postop deficits recovered in postoperative period. Overall mortality from this series was 1.33% (1 case with grade III meningioma died for intense brain swelling resistant to any treatment).

### Anatomopathological study

There were 5 (6.6%) cases classified as atypical meningioma (grade II) and 1 (1.33%) case as a grade III rhabdoid meningioma.

### Relapses

Among our cases, 10 (8 supratentorial and 2 infratentorial) were a recurrence from previous surgeries. After our surgeries, only four cases in our series were known to relapse (Table 1): two in supratentorial and two in infratentorial group. Half of them were labeled as grade II after the second surgery. It has to be considered that our series ends in 2021 and in some cases time to recurrence is still not enough.

### Illustrative case

**Figure 2:**
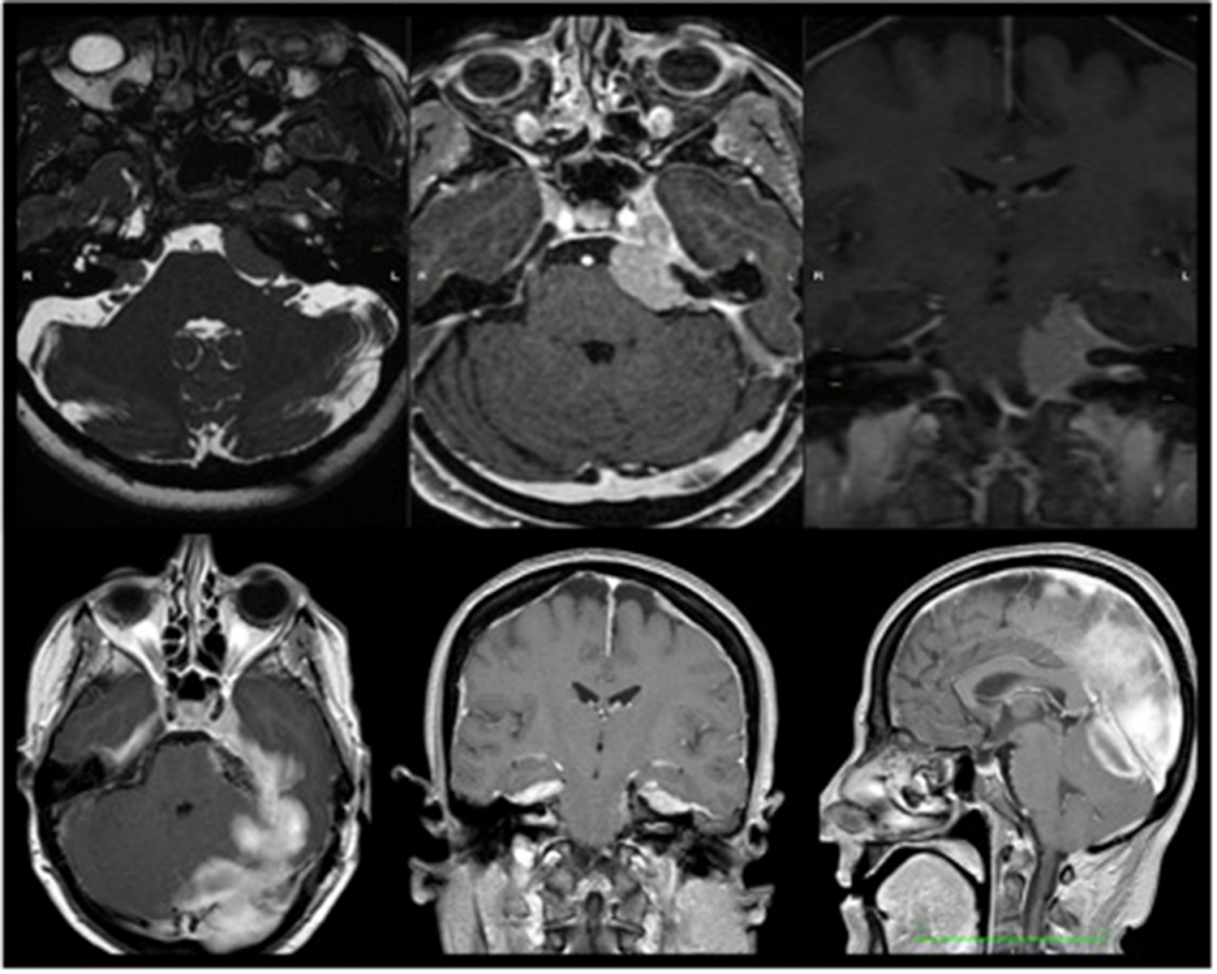
Illustrative case. Top row: preoperative images of magnetic resonance image (MRI) of the brain in sequences 3D-FIESTA, T1 + Gad axial and T1 + Gad coronal. Bottom row: postoperative image. MRI in T1 + Gad in the three planes of space.

**Figure 3:**
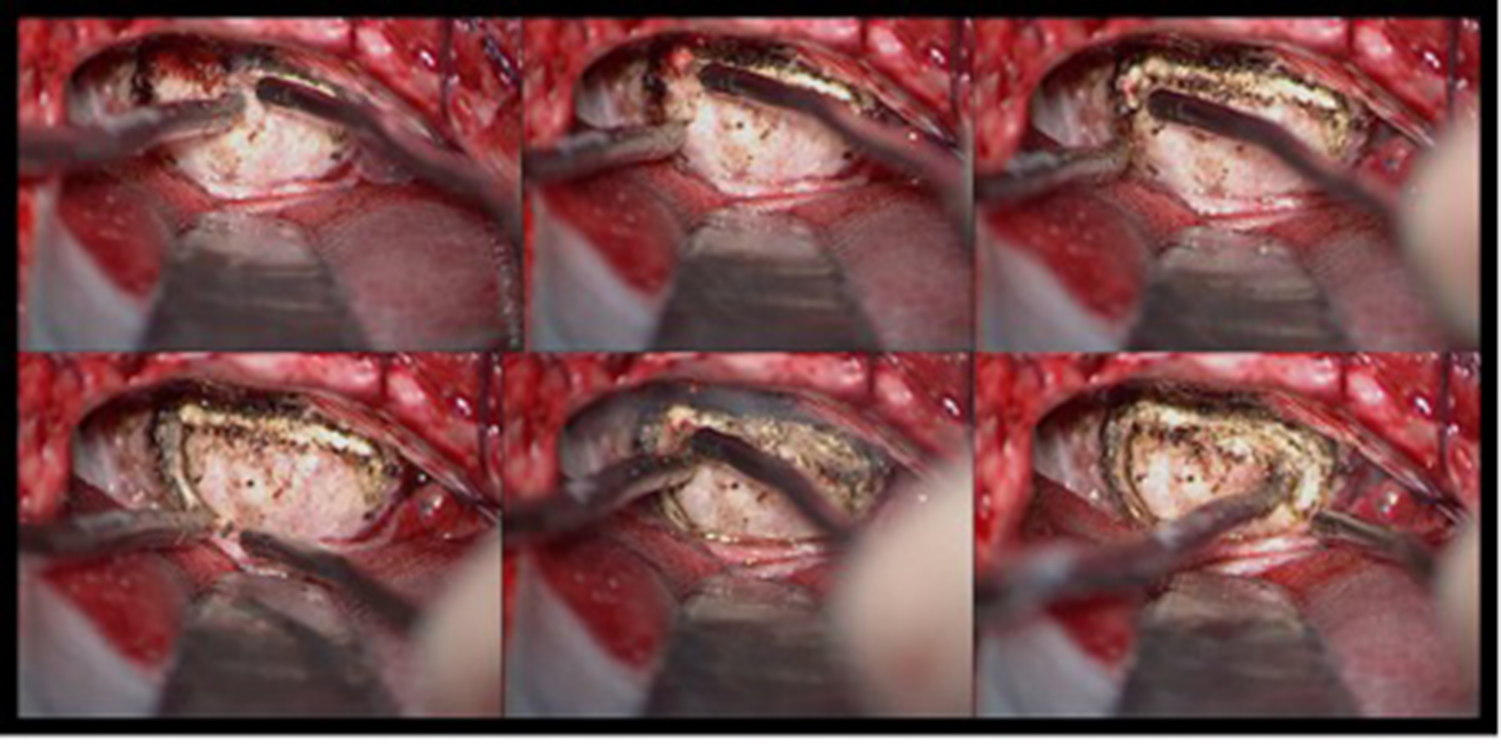
Pictures of application of the thulium laser in meningioma surgery. We appreciate in the image (look from top left to bottom right) sequential pictures of tumor capsule opening.

We present the case of a 56-year-old woman presenting with mild right hemiparesis, left deafness, and facial numbness. On the preoperative MRI scan (Figure 2), a sphenopetroclival meningioma was shown, involving superior and infratentorial departments. She was operated through a retrosigmoid craniotomy, with assistance of the 2-μm thulium laser (Figure 3). A near total resection was obtained. During surgery, a complete anatomical conservation of cranial nerves was achieved. On the postoperative MRI (Figure 2), we appreciate total resection, and a circumscribed area of parenchymal edema, at the left middle cerebellar peduncle and left cerebellar hemisphere. However, the patient presented with no neurological deficits in the postoperative examination.

## Discussion

Technology in neurosurgery has the aim of improving outcomes, helping the surgeon and limiting complications. Lasers and neurosurgery relationship is coming back now stronger than ever in our times, thanks to the many advantages of modern devices which resolve previous issues, such as the 2-μm thulium laser.

First, it allows bloodless tissue coagulation and ablation (depending on the power intensity and distance of application). Its anti-edema and vasoconstrictive potential allows for delicate cytoreduction maneuvers that facilitate exeresis, avoiding excessive bleeding in those lesions supplied by abundant arterial feeders. Second, energy is transported through thin optical fibers, which allows flexible configurations for an endoscopic or handheld device. Third, due to its particular wavelength (2 μm), it is strongly absorbed by the surrounding aqueous medium, avoiding dispersion and damage to the surrounding structures; finally, it is presented as a small device the size of a standard console, which does not usually pose space problems in the operation room. Thanks to all those treats, it constitutes a useful tool for meningioma microsurgery, helping avoid mechanical traction and continuous aspiration, facilitating capsule opening, intralesional debulking, and dissection. In addition, in case of incomplete resection, it is appropriate to safely coagulate tumor remnants and the dural implant base. We show a brief experience in the Supplementary Video.

As potential inconvenient traits, it would require a learning curve and proper carefulness by the surgeon during its application, but we have found this curve to be short and easy to undergo. It might be argued that laser would be a redundant technology, as other classical instruments, like the diathermic loop or monopolar cautery would already accomplish the same function. However, compared with it, the 2-μm thulium laser avoids electrical and thermal dispersion effects, can be applied in a more wide range of angles and positions and comes perfectly regulated for coagulation or cut. Its coagulation capacities reduce the need of bipolar forceps, so the changing of instruments gets reduced. It makes a good surgical partner to the ultrasonic aspiration device, to achieve proper debulking. In short, it is a complement to the usual instruments that offers several possibilities. Indeed, cost-effectiveness for the medical institution is favorable, as is not a very expensive tool and can be employed by several specialties.

As shown by previous studies, it causes very limited tissue penetration (around 2 mm maximum), with a cleaner and more precise cut than bipolar forceps. This is also superior to previous laser devices. The lesion that it causes can be stratified into three main areas: (a) central area or microsurgical crater, (b) honeycomb zone originated by the vaporization of cells, and (c) shrunken zone consisting of cells with pyknotic nucleus. Interestingly, the depth of the crater varied from 0.8 mm (6 W) to 1.2 mm (12 W), and in all three layers, there were no areas of microhemorrhage, confirming the focused coagulative power of the device. An *in vivo* study on murine brain performed by Katta et al. (12) highlighted how the use of 15-W thulium fiber laser allowed sharper bloodless cutting, both linear and curvilinear, with a thermal injury less than 100 µm and a removal rate of around 5 mm^3^.

As mentioned, lasers have been used in neurosurgery for different purposes. Third ventriculostomy has been performed by some groups in big series with optimal results and no damage to the basilar and related arteries (7, 9). However, for this purpose, the power should be limited to 4 W. The use of a 2-µm thulium laser has also been developed in vestibular neurinoma surgery by our group, being especially suitable for capsule opening (“V cut”), central debulking, and internal auditory canal opening. That accounts mainly for the large ones (Koos grades III and IV) and those with a cystic necrotic component, characterized by abnormal hypervascularization (8, 13, 14). Passacantilli et al. reported a 20-patients series of meningioma operated with this specific laser (10, 15, 16). Otherwise, some other laser models are currently being investigated for neurosurgical procedures, such as the 980-nm diode laser and the thulium-doped yttrium aluminum perovskite (Tm:YAP) crystal laser (17, 18). Colasanti et al. show the appliance of a modern CO_2_ laser in a variety of cases (19). As we found it more useful for hard or fibrous tissues, this group interestingly has applied laser to pial opening and final excisional bed coagulation among other surgical steps.

In our series, which comes from 2014 to 2021, we have a high representation of posterior fossa meningiomas (40 out of 75 cases). Particular attention is paid for uses arising from the tentorium (20). Some of them present as occupying the pontocerebellar angle, as the T6-T7 type [according to the classification of Yasargil modified by Al-Mefty (21)], and thus presenting symptomatology characterized by hypoacusia, tinnitus, and vertigo. Other meningiomas with subtentorial development mimicking CPA lesions are those involving the foramen lacerum, superior and inferior petrosal sinuses, sigmoid–transverse junction, and the inner dura of the acoustic porus. In those cases, a retrosigmoid or lateral suboccipital approach is usually indicated. Importantly, it is mandatory to avoid injury to the capital surrounding structures (SCA, AICA, PICA, V to XII cranial nerves), and limit the traction on brainstem structures, which could cause inadvertent microvascular disruption and major morbidity.

As a group with a broad experience in CPA tumors, we have observed that, as well as for vestibular schwannomas, the use of this laser in tentorial, CPA, and petroclival meningioma is favorable to the aforementioned objectives. We used 2-µm thulium laser under microscopic guidance: It allowed a precise cut to demarcate the area within the capsule and continue with the intralesional debulking, allowing the coagulation of microhemorrhagic spots resulting from an intraoperative break of neoangiogenic bed, thus facilitated dissection of the capsule without thermal energy transfer in nearby nerves.

In this series, that constitute the biggest in literature with 75 patients, the use of a handheld flexible thulium laser has been proved as a safe and helpful instrument for meningioma resection. In particular, we appreciated the use of a handheld flexible laser fiber appeared to be safe and easy to handle. We concluded that thulium laser does not suppose a particular risk for healthy brain or neurovascular structures around the working space. Power regulation allowed us to regulate intensity according to the particular moment requirements.

We could establish that, in our series, the degree of resection and neurological outcome were not different from previous series (20). Our impression is that handheld thulium laser fiber shortens and facilitates the duration of the steps where it is involved. Consequently, expertise in using the 2-μm thulium laser may lead to better results and shorter interventions.

Our study has several limitations: It is a longitudinal study based on a retrospective cohort and performs a descriptive analysis of the patients. Further randomized studies with a control group about surgical time, blood loss, and neurological outcome will have to be performed in the future. Also, the expansion of this laser's fields of applications (as, for instance, soft tissue dissection or different tumors, or for endonasal skull base surgery) should be explored. What is certain is that, if properly considered, many groups will progressively find different utilities for it in neurosurgery.

## Conclusion

Intracranial meningiomas can represent a neurosurgical challenge because of several factors such as their source site, the region toward which they extend, their more or less vascularized pattern, and the relationships with delicate neurovascular structures. As found in this retrospective study, the 2-μm thulium laser has proven to be a safe and useful assistant to the surgery, especially in complex cases. It has allowed us to perfect the dissection techniques and microsurgical exeresis, ensuring excellent control of bleeding and avoiding any harmful dispersion of thermal energy into the surrounding healthy tissues. Consequently, we deem it very useful and will continue to work with it.

## Data Availability

The raw data supporting the conclusions of this article will be made available by the authors, without undue reservation.
