# Supplementary material for: Safeness and efficacy of 2-µm handheld thulium laser during microsurgical resection of supratentorial and infratentorial meningiomas: Experience of a single center
Source: Front Surg. 2022 Dec 16;9:1021019. doi: 10.3389/fsurg.2022.1021019 (PMC9800793; doi:10.3389/fsurg.2022.1021019)
Supplement: Supplementary file 1 [file Table1.docx]

| **N** | **Sex** | **Age** | **Location of meningioma** | **Approach** | **Resection degree** | **Reintervention?** | **Post-op result** | **Recidive?** |
| --- | --- | --- | --- | --- | --- | --- | --- | --- |
| 5 | M | 45 | Cerebellopontine angle | Retrosigmoid | Simpson I | No | No neurological déficit / improvement |  |
| 6 | F | 45 | Cerebellopontine angle | Retrosigmoid | Simpson I | No | No neurological deficit or improvement |  |
| 7 | F | 39 | Cerebellopontine angle | Retrosigmoid | Simpson I | No | No neurological deficit or improvement |  |
| 8 | F | 43 | Cerebellopontine angle | Retrosigmoid | Simpson II | No | No neurological deficit or improvement |  |
| 9 | M | 68 | Cerebellopontine angle | Retrosigmoid | Simpson I | No | No neurological deficit or improvement |  |
| 10 | F | 65 | Posterior Petrous bone | Retrosigmoid | Simpson IV | No | VI, VII and VIII cranial nerve deficit |  |
| 11 | F | 28 | Posterior foramen lacerum | Retrosigmoid | Simpson I | No | No neurological deficit or improvement |  |
| 12 | F | 70 | Tentorial / Posterior petrous bone | Retrosigmoid | Simpson III | No | Cranial nerve deficit |  |
| 13 | F | 47 | Petroclival | ELITE | Simpson IV | No | No neurological deficit or improvement | yes, 2 yr WHO I |
| 14 | M | 63 | Petroclival | Retrosigmoid | Simpson III | No | Cranial nerve deficit (VII, V) |  |
| 15 | F | 68 | Petroclival | Retrosigmoid | Simpson IV | No | No neurological deficit or improvement |  |
| 16 | F | 68 | Petroclival | Kawase | Simpson III | No | Consciounsens level transient impairment |  |
| 17 | F | 34 | Petroclival | Retrosigmoid | Simpson II | Yes | No neurological deficit or improvement |  |
| 18 | F | 45 | Petroclival | Retrosigmoid | Simpson II | No | No neurological deficit or improvement |  |
| 19 | M | 50 | Petroclival | Retrosigmoid | Simpson I | No | Major neurological deficit |  |
| 20 | F | 74 | Petroclival | Retrosigmoid | Simpson II | No | Mild IV cranial pair deficit |  |
| 21 | F | 56 | Petroclival | Retrosigmoid | Simpson II | No | Mild VII cranial pair deficit |  |
| 22 | M | 68 | Petroclival | Retrosigmoid | Simpson IV | No | Cranial nerve deficit (VII, V, IX) |  |
| 23 | F | 32 | Tentorial / Posterior petrous bone | Retrosigmoid | Simpson II | No | No neurological deficit or improvement | yes, 8 yrs WHO I |
| 24 | F | 62 | Tentorial / Posterior petrous bone | Retrosigmoid | Simpson II | No | No neurological deficit or improvement |  |
| 25 | F | 80 | Tentorial / Posterior petrous bone | Retrosigmoid | Simpson I | Yes | No neurological deficit or improvement |  |
| 26 | F | 76 | Spheno-petroclival | Retrosigmoid | Simpson IV | No | No neurological deficit or improvement |  |
| 34 | F | 52 | Petroclival | Retrosigmoid and inverse Kawase | Simpson II | No | Cranial nerve deficit (VI, VI) |  |
| 35 | M | 65 | Petroclival | Retrosigmoid | Simpson I | No | Mild VII cranial pair deficit |  |
| 36 | F | 63 | Cerebellopontine angle | Retrosigmoid | Simpson I | No | No neurological deficit or improvement |  |
| 37 | F | 44 | Petroclival | Kawase | Simpson III | No | Motor and cranial nerve transient deficit |  |
| 45 | F | 41 | Cerebellopontine angle | Retrosigmoid | Simpson II | No | No neurological deficit or improvement |  |
| 46 | M | 57 | Petroclival | Retrosigmoid | Simpson III | No | No neurological deficit or improvement |  |
| 47 | F | 55 | Foramen magnum | ELITE | Simpson IV | No | No neurological deficit or improvement |  |
| 48 | M | 35 | Tentorial | Supracerebellar infratentorial | Simpson II | No | No neurological deficit or improvement |  |
| 49 | F | 43 | Posterior foramen lacerum | ELITE | Simpson III | No | Motor and cranial nerve transient deficit |  |
| 51 | F | 64 | Cerebellopontine angle | Retrosigmoid | Simpson I | No | No neurological deficit or improvement |  |
| 56 | F | 65 | Cerebellopontine angle | Retrosigmoid | Simpson I | No | No neurological deficit or improvement |  |
| 57 | M | 46 | Foramen magnum | Suboccipital | Simpson I | No | No neurological deficit or improvement |  |
| 61 | F | 65 | Cerebellopontine angle | Retrosigmoid | Simpson II | No | No neurological deficit or improvement |  |
| 63 | F | 76 | Petroclival | Retrosigmoid | Simpson II | No | Mild VIII cranial nerve deficit |  |
| 64 | F | 38 | Cerebellopontine angle | Retrosigmoid | Simpson II | No | No neurological deficit or improvement |  |
| 66 | F | 50 | Foramen magnum | ELITE | Simpson IV | No | No neurological deficit or improvement |  |
| 68 | F | 50 | Cerebellopontine angle | Retrosigmoid | Simpson II | No | No neurological deficit or improvement |  |
| 70 | M | 37 | Cerebellopontine angle | Retrosigmoid | Simpson II | No | Mild VII cranial pair deficit |  |
| 72 | F | 50 | Foramen magnum | ELITE | Simpson II | No | No neurological deficit or improvement |  |
| 73 | F | 56 | Spheno-petro-clival | Retrosigmoid | Simpson IV | No | No neurological deficit or improvement |  |
| 75 | M | 62 | Cerebellopontine angle | Retrosigmoid | Simpson IV | No | Verbal impairment |  |

**Supplementary material 2:** Individual patient-data of infratentorial meningioma cases.
