# Supplementary material for: Safeness and efficacy of 2-µm handheld thulium laser during microsurgical resection of supratentorial and infratentorial meningiomas: Experience of a single center
Source: Front Surg. 2022 Dec 16;9:1021019. doi: 10.3389/fsurg.2022.1021019 (PMC9800793; doi:10.3389/fsurg.2022.1021019)
Supplement: Supplementary file 2 [file Table2.docx]

| **N** | **Sex** | **Age** | **Location of meningioma** | **Approach** | **Resection degree** | **Reintervention?** | **Post-op result** | **Recidive?** |
| --- | --- | --- | --- | --- | --- | --- | --- | --- |
| 1 | F | 49 | Medium cranial fosa | Right temporal craniotomy, far lateral triangle drilling | Simpson I | No | No neurological deficit or improvement | yes, 1 yr WHO II |
| 2 | F | 59 | Medium cranial fosa - Anterior clinoid process | Orbitopterional craniotomy | Simpson I | No | No neurological deficit or improvement |  |
| 3 | F | 81 | Medium cranial fosa - Anterior clinoid process | Pterional craniotomy | Simpson III | No | VI cranial nerve deficit |  |
| 4 | F | 67 | Medium cranial fosa | Orbitopterional craniotomy | Simpson III | Yes | No neurological deficit or improvement | yes, 2 yr WHO II |
| 27 | M | 78 | Frontal parasagital | Ad-hoc craniotomy | Simpson II | No | No neurological deficit or improvement |  |
| 28 | M | 71 | Sphenoidal wing | Pterional craniotomy | Simpson II | No | Mild left hemiparesis |  |
| 29 | F | 34 | Intraventricular | Ad-hoc craniotomy | Simpson IV | No | Right hemiparesis, disartria and memory impairment |  |
| 30 | M | 72 | Parafalcine | Ad-hoc craniotomy | Simpson III | No | No neurological deficit or improvement |  |
| 31 | F | 63 | Medium cranial fosa | Pterional craniotomy | Simpson I | No | Trasncoortical disfasia |  |
| 32 | F | 59 | Tubercullum sellae | Pterional craniotomy | Simpson I | No | Left hemiparesis, motor and sensory afasia |  |
| 33 | M | 62 | Orbital roof | Frontobasal craniotomy (eyebrow incission) | Simpson I | Yes | No neurological deficit or improvement |  |
| 38 | M | 78 | Parafalcine | Ad-hoc craniotomy | Simpson I | Yes | No neurological deficit or improvement |  |
| 39 | F | 81 | Sphenoidale | Pterional craniotomy | Simpson II | No | No neurological deficit or improvement |  |
| 40 | F | 37 | Petro-sfeno-clival | Kawase | Simpson IV | Yes | Left hemiparesis |  |
| 41 | F | 46 | Temporo-basal / Tentorial | Ad-hoc craniotomy | Simpson I | No | No neurological deficit or improvement |  |
| 42 | M | 75 | Parafalcine | Ad-hoc craniotomy | Simpson I | No | No neurological deficit or improvement |  |
| 43 | F | 68 | Parafalcine | Ad-hoc craniotomy | Simpson II | No | No neurological deficit or improvement |  |
| 44 | F | 77 | Convexity (rolandic) | Ad-hoc craniotomy | Simpson I | No | No neurological deficit or improvement |  |
| 50 | M | 56 | Temporo-basal / Tentorial | Pterional craniotomy | Simpson IV | Yes | No neurological deficit. Complicated with sepsis |  |
| 52 | M | 63 | Parafalcine | Ad-hoc craniotomy | Simpson I | No | No neurological deficit or improvement |  |
| 53 | M | 53 | Anterior clinoid process | Pterional craniotomy | Simpson IV | No | III cranial nerve déficit |  |
| 54 | F | 75 | Parafalcine | Ad-hoc craniotomy | Simpson II | No | Speech impairment |  |
| 55 | M | 64 | Medium cranial fosa + Parietal extension | Pterional craniotomy | Simpson II | Yes | No neurological deficit or improvement |  |
| 58 | F | 47 | Medium cranial fosa - Anterior clinoid process | Pterional craniotomy | Simpson I | No | No neurological deficit or improvement |  |
| 59 | F | 43 | Spheno-orbital | Pterional craniotomy | Simpson II | No | No neurological deficit or improvement |  |
| 60 | F | 45 | Parafalcine | Ad-hoc craniotomy | Simpson II | No | No neurological deficit or improvement |  |
| 62 | F | 78 | Intraorbital | Frontobasal craniotomy (eyebrow incission) | Simpson IV | Yes | No neurological deficit or improvement |  |
| 65 | F | 83 | Anterior and Medium cranial fossa (spheno-orbital, infratemporae, intranasal) | Endoscopic (trasnasal) + Pterionale | Simpson IV | No | No neurological deficit or improvement |  |
| 67 | F | 70 | Parafalcine | Ad-hoc craniotomy | Simpson I | No | No neurological deficit or improvement |  |
| 69 | F | 47 | Parafalcine | Ad-hoc craniotomy | Simpson I | No | Left limb paresis |  |
| 71 | M | 58 | Spheno-cavernous | Pterional craniotomy | Simpson IV | Yes | No neurological deficit or improvement |  |
| 74 | F | 56 | Sphenoidal wing | Pterional craniotomy | Simpson II | No | No neurological deficit or improvement |  |

**Supplementary material 1:** Individual patient-data of supratentorial meningioma cases.
